# Bronchiolitis Severity Affects Blood Count and Inflammatory Marker Levels: A Real-Life Experience

**DOI:** 10.3390/v17010077

**Published:** 2025-01-09

**Authors:** Antonella Gambadauro, Salvatore Mollica, Emanuela Rosa, Federica Xerra, Alessandra Li Pomi, Mariella Valenzise, Maria Francesca Messina, Agata Vitale, Eloisa Gitto, Malgorzata Wasniewska, Giuseppina Zirilli, Sara Manti

**Affiliations:** 1 Pediatric Unit, Department of Human Pathology in Adult and Developmental Age “Gaetano Barresi”, University of Messina, Via Consolare Valeria 1, 98124 Messina, Italy; salvo.mollica@gmail.com (S.M.); federicaxerra@gmail.com (F.X.); mariella.valenzise@unime.it (M.V.); francesca.messina@unime.it (M.F.M.); agata.vitale@polime.it (A.V.); malgorzata.wasniewska@unime.it (M.W.); giuseppina.zirilli@unime.it (G.Z.); sara.manti@unime.it (S.M.); 2 Faculty of Medicine and Surgery, University of Messina, Piazza Pugliatti 1, 98122 Messina, Italy; emanuelarosamed@gmail.com; 3 Neonatal and Pediatric Intensive Care Unit, Department of Human Pathology in Adult and Developmental Age “Gaetano Barresi”, University of Messina, Via Consolare Valeria 1, 98124 Messina, Italy; eloisa.gitto@unime.it

**Keywords:** bronchiolitis, inflammation, severity score, pediatric intensive care unit, infants

## Abstract

Background: Bronchiolitis is the most common cause of lower respiratory tract infection (LRTI) in the first year of life. We analyzed the association between complete blood count (CBC), c-reactive protein (CRP), and novel inflammatory indexes (NLR, PLR, MLR, ELR, LMR, NPR, LPR, LNR, PNR, SII, SIRI) in predicting bronchiolitis severity at hospital admission. Methods: We retrospectively collected data from 95 infants hospitalized for bronchiolitis in a third-level hospital during three epidemic seasons. Five outcomes of severity were analyzed: BRAS; pediatric intensive care unit (PICU) admission; ventilatory support; intravenous (IV) rehydration; and length of stay (LOS). Results: Lower age and weight at admission were statistically associated with four of the five severity outcomes. Prolonged LOS (≥6 days) was associated with high values of total white blood cells, lymphocytes, and eosinophils. Only three inflammatory indexes (PLR, MLR, and PNR) showed a significant association with one outcome (prolonged LOS). A new index (RBC/AiW/1000) was statistically associated with each severity outcome for a value > 350. Conclusions: We proposed a comprehensive analysis of the association between CBC, CRP, and novel inflammatory indexes and bronchiolitis severity. RBC/AiW/1000 could represent a future predictive marker of disease severity at hospital admission in infants with bronchiolitis.

## 1. Introduction

Viral bronchiolitis constitutes the most common cause of lower respiratory tract infection (LRTI) in infants in their first year of life, leading to a significant economic burden worldwide [1]. Among viral infections, *Respiratory Syncytial Virus* (RSV) is the most frequently detected, followed by *Rhinovirus*, *Parainfluenza* virus, and *Metapneumovirus* [1]. The diagnosis of bronchiolitis is based on clinical history and physical examination [2]; however, in children hospitalized for moderate to severe disease, laboratory tests are usually performed to identify the presence of dehydration or sepsis. Hospitalization is suggested mainly in infants younger than 3 months or with risk factors, such as prematurity, bronchopulmonary dysplasia (BPD), congenital heart diseases, and immunodeficiency [1,2].

Numerous clinical aspects have been studied to identify a possible correlation with bronchiolitis severity, resulting in the adoption of several clinical severity scores. However, most of these instruments are based on auscultation skills and clinical evaluations that, being subjective, can affect validity. In their systematic review, Rodriguez et al. [3] identified the most reported items in defining the severity of bronchiolitis and described as the best evidence-based available scoring system the bronchiolitis risk of admission score (BRAS) [4]. This score is based on anamnestic parameters (duration of symptoms and age at presentation) and vital signs (respiratory rate, heart rate, and oxygen saturation) [4], and it is frequently used in clinical practice and literature research.

The intersubject variability in determining some items reported in clinical scores has increased the interest in objective measurements, such as laboratory data. Viral respiratory infections significantly impact the immune system, perturbating the expression of blood cells and the host inflammatory status [5]. It is reasonable to hypothesize that some early laboratory markers can predict the disease severity in infants with bronchiolitis. Some studies reported a correlation between lymphopenia and bronchiolitis severity [6,7,8], while eosinophil and platelet count seem to be not associated with a negative disease outcome [9,10]. High c-reactive protein (CRP) levels seem to be associated with prolonged hospitalization, pediatric intensive care unit (PICU) admission, and the requirement for oxygen supplementation [11]. Recently, specific blood markers obtained from routine complete blood counts (CBCs), such as monocyte-to-lymphocyte ratio (MLR) and neutrophil-to-lymphocyte ratio (NLR), were suggested as potential biomarkers for the identification of infants with RSV infection at major risk for severe outcomes [12]. Moreover, novel inflammatory markers have been analyzed to predict morbidity and mortality in different conditions [13,14].

To date, a comprehensive analysis of early changes in CBC, CRP, and novel inflammatory markers in determining bronchiolitis severity in infants and children is not reported in the literature. The purpose of our study was to describe the laboratory picture of infants with bronchiolitis in relationship to clinical severity outcomes at admission in a pediatric ward (PW) or a PICU of a third-level hospital.

## 2. Materials and Methods

### 2.1. Study Design and Participants

We performed a monocentric, retrospective cohort study analyzing data from infants aged < 12 months admitted with a diagnosis of bronchiolitis to the Pediatric Unit or the Pediatric Intensive Care Unit (PICU) of the “G. Martino” Hospital, Messina (Italy). We collected data over three epidemic seasons, from October to May 2021 to 2024. Data were extracted using patients’ electronic medical records through the hospital platform. However, only patients who performed nasal swabs for microbiological analysis were initially selected for the study.

Inclusion criteria were children aged < 12 months with a clinical diagnosis of bronchiolitis. Bronchiolitis was defined as an LRTI characterized by respiratory distress, cough, wheezing, and/or crackles [15]. For the purpose of our study, we included both the first and subsequent episodes of bronchiolitis. Only patients with viral infections were included in our study.

Exclusion criteria were children with co-morbidities (chronic neurological or conditions, hemodynamically significant congenital heart diseases, chronic lung diseases, immunodeficiency, blood disorders, trisomy 21), and being extremely preterm (<28 weeks). Moreover, we excluded children receiving treatment with systemic corticosteroids, antibiotics, and antivirals before the hospital admission. Bacterial infection at nasal swabs was also considered an exclusion criterion.

### 2.2. Study Procedures

We retrospectively collected anamnestic (age, gender, weight, presence of co-morbidities, gestational age, duration of symptoms) and clinical data (respiratory rate, heart rate, oxygen saturation). We calculated BRAS [4] according to the patients’ clinical status at admission. At admission, all children performed laboratory tests for CBC, CRP, and nasal swabs for pathogen detection using the Filmarray system (multiplex polymerase chain reaction (PCR) system). The respiratory panel for viral and bacterial infections (Filmarray system) comprised: *Adenovirus*; *Coronavirus* 229E, HKU1, NL63, and OC43; *Severe Acute Respiratory Syndrome Coronavirus 2* (SARS-CoV-2); Influenza A subtypes H1 and H3; Influenza B; *Metapneumovirus*; *Parainfluenza* virus type 1, type 2, type 3, and type 4; RSV; *Rhinovirus*; *Enterovirus*; *Bordetella pertussis*; *Chlamydia pneumoniae*; *Mycoplasma pneumoniae; Haemophilus influenzae; Streptococcus pneumoniae*. CBC was measured by flow cytometry, while CRP quantitative determination was detected by immunoturbidimetry.

We obtained the following inflammatory indexes from the absolute cell counts performed at admission: NLR (neutrophils/lymphocytes ratio), PLR (platelets/lymphocytes ratio), MLR (monocytes/lymphocytes ratio), ELR (eosinophils/lymphocytes ratio), LMR (lymphocytes/monocytes ratio), NPR (neutrophils/platelets ratio), LPR (lymphocytes/platelets ratio), LNR (lymphocytes/neutrophils ratio), PNR (platelets/neutrophils ratio), SII (systemic immune-inflammation index, NLR*platelets), and SIRI (systemic inflammation response index, MLR*neutrophils) [13,14]. We also calculated two new indexes: the ratio between age in weeks and hemoglobin levels (AiW/Hb); and the ratio between red blood cells and age in weeks divided by 1000 (RBC/AiW/1000).

To define the severity of bronchiolitis, we considered the following data: BRAS at admission; admission to PICU; need for supplementary oxygen (oxygen mask or nasal cannula), high flow nasal cannula (HFNC), continuous positive air pressure (CPAP), or non-invasive ventilation (NIV); intravenous (IV) fluids administration; and length of stay (LOS).

### 2.3. Statistical Analysis

For the analysis of the descriptive data, we used percentages (%), ranges, mean, and standard deviation (SD), or median and interquartile range (IQR). We analyzed the demographic and clinical characteristics of our sample, such as age, preterm birth, gender, weight at admission, viral detection at Filmarray, admission to PICU, IV rehydration, ventilatory support, LOS in the hospital, and BRAS at admission.

We derived the mean and SD of each blood parameter, represented by blood white and red cell count, platelet count, neutrophils, lymphocytes, eosinophils, monocytes, hemoglobin, and CRP. We also calculated mean and SD of novel inflammatory indexes (NLR, PLR, MLR, ELR, LMR, NPR, LPR, LNR, PNR, SII, and SIRI) as well as AiW/Hb and RBC/AiW/1000.

Student’s t-distribution was used to assess the association between blood parameters (including inflammatory indexes) and severity outcomes during hospitalization. When a statistical association between blood parameters and severity outcomes were found, we established a cut-off value and calculated sensitivity, specificity, positive and negative predictive values (PPV and NPV), Odds Ratio (OR) and Relative Risk (RR) associated with that, to evaluate its possible prognostic role.

The statistical analyses were performed using Microsoft Excel (2409 version) and MedCalc (2024 MedCalc Software Ltd., Ostend, Belgium). Statistical significance was considered *p* < 0.05.

### 2.4. Ethics

This study was conducted in accordance with the Helsinki declaration [16]. Informed consent was not required due to the retrospective nature of the study.

## 3. Results

### 3.1. Demographic and Clinical Characteristics

**Figure 1:**
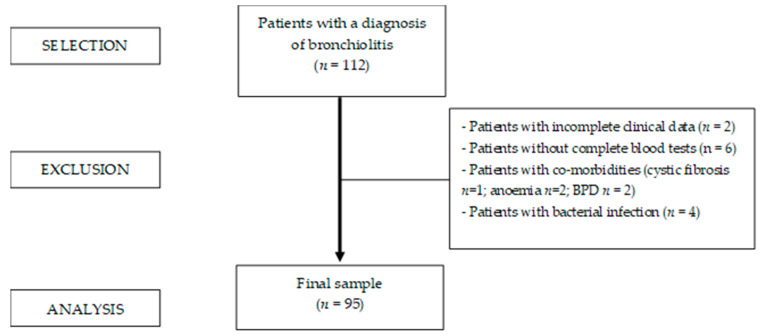
Flowchart of patients included in the study.

Among the 112 infants initially enrolled in the study, we included 95 patients with a median age of 12 weeks (IQR 8–20; F = 42%) (Figure 1). In total, 12% of our patients were born preterm, and 6% had co-morbidities. A viral respiratory infection was reported in all patients, and the pathogens detected by Filmarray system were RSV (82%), *Rhinovirus* (26%), *Metapneumovirus* (13%), *Adenovirus* (4%), and other pathogens (19%). Considering the severity outcomes during hospitalization, 39% of patients needed PICU admission, 65% required ventilatory support or oxygen supplementation, and 59% needed IV rehydration. The BRAS median value at admission was 2 (IQR 2–4), and the median LOS was 8 (IQR 6–10). The demographic and clinical characteristics are reported in Table 1.

### 3.2. Laboratory Parameters and Inflammatory Indexes

We divided our patients into two groups based on five items (PICU admission, use of ventilatory support, IV rehydration, LOS ≥ 6 days or ≤5 days, BRAS ≥ 3 or ≤2) and evaluated if there were any significant statistical differences among the pairs of groups regarding demographic data, CBC values, CRP, or novel inflammatory markers.

Comparing the group of patients who required PICU admission (n = 37) with the group of patients who did not require it (n = 58), there was a statistically significative difference between the age in weeks and the weight at admission (*p* < 0.001), with inferior values for patients admitted to PICU; no statistical difference was found for gestational age at birth and gender. By analyzing CBC values, higher values of hemoglobin and lower values of red blood cells were significantly connected to PICU admission (*p* < 0.05). A value of RBCs ≤ 4,050,000/mmc was found to have NPV 71% and RR 1.5. AiW/Hb < 0.85 and RBC/AiW/1000 > 350 were associated with PICU admission, with a similar NPV (83 and 82, respectively) and an RR equal to 2.9 and 2.2, respectively. No difference was found for CRP and novel inflammatory markers. Data are synthesized in Table 2 and Table 3.

Comparing the group of patients who required ventilatory support (n = 62) with the group of patients who did not require it (n = 33), there was a statistically significative difference between the age in weeks and the weight at admission (*p* < 0.001), with inferior values for patients who required ventilatory support; no statistical difference was found for gestational age at birth and gender. By considering CBC values, higher values of red blood cells were significantly related to no need for ventilatory support (*p* < 0.05). Considering the population requiring ventilatory support, a value of RBCs ≤ 4,300,000/mmc had a PPV of 72% and a RR of 1.4. Moreover, RBC/AiW/1000 > 350 and AiW/Hb < 1 had a PPV of 83% and 81%, respectively (with a RR of 2.7 and 2.3, respectively). No difference was found for CRP and novel inflammatory markers. Data are synthesized in Table 4 and Table 5.

Comparing the group of patients who required IV fluid administration (n = 56) with the group of patients who did not require it (n = 39), there was a statistically significative difference between the age in weeks and the weight at admission (*p* < 0.001), with inferior values for patients that required intravenous fluids; moreover, higher values of red blood cells were significantly related to no need for IV fluids (*p* < 0.05). Considering the population requiring IV fluids, a value of RBCs ≤ 4,300,000/mmc had a PPV of 70% and an RR of 1.6. RBC/AiW/1000 > 350 and AiW/Hb < 1 had a PPV of 78% and 79%, respectively (with an RR of 2.5 and 2.6, respectively). No difference was found for CPR and novel inflammatory markers. Data are synthesized in Table 6 and Table 7.

Comparing the group of patients with LOS ≥ 6 days (n = 76) with the group of patients with LOS ≤ 5 days (n = 19), there was any significant statistical difference between the age in weeks, weight at admission, gestational age, and gender between the two groups. By analyzing CBC values, higher values of total white blood cells, lymphocytes, and eosinophils were related to a prolonged LOS (*p* < 0.05). Specifically, WBC ≥ 9300/mmc had higher sensitivity (72%), a PPV of 90%, and an RR of 2.3; lymphocytes ≥ 4900/mmc had higher specificity (79%), PPV of 91%, and an RR of 2.7; eosinophils ≥ 110/mmc had a PPV of 89% and an RR of 2.0. We found an association between LOS ≥ 6 days and RBC/AiW/1000 > 350, with a PPV of 88% and an RR of 1.8. By considering the inflammatory indexes, lower values of PLR, MLR, and PNR were found in the group of patients with a prolonged LOS (*p* < 0.05), with an RR of 1.4, 1.4, and 1.2, respectively (considering as cut-off values ≤ 112, ≤0.15, and ≤99, respectively). Data are synthesized in Table 8 and Table 9.

Comparing the group of patients with a BRAS ≥ 3 (n = 40) with the group of patients with a BRAS ≤ 2 (n = 55), there was a significant statistical difference between the age in weeks and the weight at admission (*p* < 0.001), with lower values in patients with higher a BRAS; no statistical difference was found for gestational age at birth and gender. By considering CBC values, higher values of hemoglobin were significantly related to a BRAS ≥ 3 (*p* < 0.05), with an NPV of 61% and an RR of 1.1 for a hemoglobin cut-off value > 11.2 g/dL. For BRAS ≥ 3, AiW/Hb < 0.85 had a PPV of 65%, an NPV of 77%, and an RR of 2.6, while RBC/AiW/1000 > 350 had a PPV of 62%, an NPV of 80%, and an RR of 2.2. No difference was found for CPR and novel inflammatory markers. Data are synthesized in Table 10 and Table 11.

## 4. Discussion

Our study provides a comprehensive analysis of the early changes in CBC, CRP, and novel inflammatory markers in children with bronchiolitis. We collected data over three epidemic seasons of children hospitalized in the PW or PICU of a third-level hospital to assess the relationship between early laboratory findings and severity outcomes.

Lower age and weight at admission significantly impacted disease severity, especially for four of the five items (PICU admission, ventilatory support, IV fluids administration, and BRAS). In our cohort, these two demographic variables did not present association with LOS. Previous studies have found that younger infants with bronchiolitis were at higher risk of needing oxygen supplementation and PICU admission [17,18]. Moreover, the presence of different severity profiles of bronchiolitis was proposed in the analysis of affected patients, in which younger infants (especially those aged < 6 months) had the most severe clinical presentation and increased incidence of respiratory distress [19,20]. This age-related susceptibility could be associated with different immune responses observed in younger infants: in children < 6 months of age, decreased levels of interferon (IFN)-γ, IFN-γ–inducible protein (IP)-10, and eotaxin were linked to higher rates of hospitalization, while in children > 6 months of age higher levels of IFNs were found and were related to lower rates of hospitalization [21,22]. The other demographic variables analyzed in our study (i.e., gestational age at birth and gender) were not associated with disease severity. In fact, in the PICU admission group, the gestational age was higher than that of the other group, but it was not statistically relevant (*p* = 0.23). Among our 11 patients with a history of prematurity, 3 of them (27%) needed PICU admission, while 8 of them (73%) did not require PICU admission, without any relevant statistical difference between the two groups (*p* = 1).

The analysis of CBC revealed that several parameters may predict disease severity in infants hospitalized with bronchiolitis. High values of hemoglobin and/or low values of RBCs were linked to more severe disease, particularly considering PICU admission, ventilatory support, IV fluid administration, and a BRAS ≥ 3. Our result was in contrast with a previous study that described an association between anemia (defined by a hemoglobin level < 10 g/dL) and a higher use of CPAP as well as prolonged respiratory support in infants with viral bronchiolitis [23]. However, this study only analyzed infants younger than 16 weeks and with heterogeneous conditions (e.g., 15% were born premature, 5% had cardiological diseases, 5% had a diagnosis of anemia before the bronchiolitis onset) [23]. Conversely, in our analysis, we reported children aged < 12 months without important co-morbidities, which could affect the interpretation of the hematological results. Iron deficiency anemia was considered protective for viral infections in infants because high iron concentration facilitates viral replication. In fact, iron and hemoglobin reduction during viral infection is considered a physiological reaction of the immune system to contrast viral replication [24,25]. It could be supposed that a high hemoglobin concentration may predict the low ability of the immunological system in some infants to respond early to viral infections, increasing the disease severity. However, further studies are needed to clarify this issue. Viral infections may also compromise the morphology, deformability, and aggregability of RBCs, reducing their count in contrast to bacterial disease [26,27]. The presence of low values of RBCs could be interpreted as an early marker of viral infection.

Due to the statistically significant association that emerged during our research, we calculated two new parameters: AiW/Hb and RBC/AiW/1000. AiW/Hb < 0.85 was significantly associated with PICU admission and BRAS ≥ 3, AiW/Hb < 1 was significantly associated with ventilatory support and IV rehydration, and RBC/AiW/1000 > 350 was significantly associated with all the five severity outcomes (PICU admission, ventilatory support, IV fluids administration, LOS ≥ 6 days, and BRAS ≥ 3). These two new indexes may identify an increased risk of severe disease in relationship to younger age and higher values of hemoglobin and/or RBC count. Further studies are required to assess the validity of these indexes.

In our cohort, we showed that high values of total white blood cells, lymphocytes, and eosinophils are associated with a prolonged LOS (≥6 days). These parameters were not significantly related to the other severity outcomes. The presence of high values of leucocytes and lymphocytes is in line with the diagnosis of viral infection in our patients. Viruses induce the production of IFN-γ, which stimulates the immune response by activating B and T lymphocytes and inhibiting viral replication [28]. However, other studies in infants with severe bronchiolitis have described the association with a specific viral agent and lymphopenia. Matera et al. described a correlation between low lymphocyte count and bronchiolitis severity, which was statistically significant related to a worse clinical severity score, a more frequent need for oxygen supplementation, prolonged hospitalization, and more frequent ICU admission. However, these authors described how the low lymphocyte count was mostly associated with RSV infection, which was prevalent in their sample [8]. RSV seems to induce lymphopenia by increasing lymphocyte apoptosis in infants with bronchiolitis [29]. Moreover, in infants with severe RSV infection, neutrophils create lung inflammatory infiltrates, but whether these cells contribute or not to determine the disease severity is still debated [30,31]. For our study, we did not analyze the impact of each virus on the blood count, also considering the wide viral heterogeneity of our cohort. Eosinophilia was previously described in association with prolonged hospitalization and with increased prevalence of mechanical ventilation [32]. Eosinophils may induce bronchoconstriction due to mucus secretion, cellular desquamation, and airway hyperresponsiveness [33,34]. These phenomena may enhance disease severity and predispose to developing wheezing and asthma in children [35].

In recent years, the literature research has analyzed the role of novel inflammatory indexes derived from blood cell counts as early predictors of severity outcomes in several inflammatory diseases, such as lung infections, cardiovascular diseases, and sepsis [13,14]. To the best of our knowledge, only two studies have already investigated their role in infants with bronchiolitis, and limited markers were considered in these analyses with contrasting results. A single-centre retrospective study on children aged < 2 years hospitalized with moderate-to-severe bronchiolitis (n = 155) did not find any statistically significant association between bronchiolitis severity and NLR or SII values. However, in this study, the disease severity was evaluated by considering only LOS and oxygen supplementation, and patients who required mechanical ventilation were not included in the final analysis [36]. The second one was a single-centre prospective study on children aged < 2 years hospitalized with bronchiolitis (n = 74). This original research showed that neutrophil count, NLR, and SII values increased significantly with bronchiolitis severity, with SII reaching the highest area under the curve (AUC) in predicting patients with high disease severity [37]. Our study did not find any significant association between disease severity and NLR or SII. Each severity outcome did not correlate with the analyzed novel inflammatory indexes, except for prolonged LOS and lower values of PLR, MLR, and PNR. These data must require future assessments to determine their validation and their possible use in the early prediction of bronchiolitis severity in infants.

Our study has some limitations. First, this was a single-centre study, affecting the generalizability of the results. Secondly, this was a retrospective study and potentially confounded by selection bias. However, we carefully checked the inclusion/exclusion criteria in our sample, and our selection method was rigorous. Thirdly, we did not analyze the influence of each pathogen on the blood parameters, due to the excessively different prevalence of each virus in our sample and the high prevalence of co-infection (Table 1). Fourthly, we did not include a control group. Lastly, we did not include the evaluation of the treatment provided during the hospitalization, which may have a positive or negative impact on the severity of the outcomes. Further studies, even multi-centre and prospective, may be helpful to increase the scientific value of our results.

## 5. Conclusions

Our study aimed to evaluate the association between CBC and novel inflammatory indexes with the severity of bronchiolitis at hospital admission. We did not find any association between novel inflammatory indexes and bronchiolitis severity, except for LOS and lower PLR, MLR, and PNR values. CBC may be used as a prognostic tool for assessing the severity of the disease in infants hospitalized with bronchiolitis. The most important CBC parameters reported associated with disease severity were RBCs, hemoglobin, white blood cells, lymphocytes, and eosinophils. Two new indexes, AiW/Hb and RBC/AiW/1000, were proposed in our study. Particularly, RBC/AiW/1000 > 350 showed a statistically significant association with each severity outcome and could represent a future blood biomarker for predicting the severity of bronchiolitis in hospitalized infants. It would be helpful to conduct further studies to explore and validate the potential of the identified biomarkers for application in clinical practice.

## Figures and Tables

**Table 1:** Demographic and clinical characteristics of our cohort (n = 95) *.

Age (weeks) *Range*	12 (8, 20) 2–52
Gender	
Males Females	55 (58%) 40 (42%)
Preterm birth	
Yes No	11 (12%) 84 (88%)
Weight at admission (gr)	5670 (4535, 7085)
*Range*	2200–14,950
Co-morbidities	6 (6%)
Congenital adrenal hyperplasia Ependymal cyst Cow’s milk allergy Mild ventricular septal defect Congenital hip dysplasia	1 (1%) 1 (1%) 1 (1%) 2 (2%) 1 (1%)
Filmarray (multiplex PCR system)	
RSV, total RSV, mono-infection Rhinovirus Metapneumovirus Adenovirus SARS-CoV-2 Others (Influenza, Parainfluenza, Enterovirus, Coronavirus OC43) Co-infections	78 (82%) 54 (57%) 25 (26%) 12 (13%) 4 (4%) 1 (1%) 17 (18%) 30 (32%)
PICU admission (yes)	37 (39%)
Ventilatory support (yes)	62 (65%)
Oxygen mask or nasal cannula High flow nasal cannula Continuous positive airway pressure Mechanical ventilation	32 (34%) 30 (32%) 0 (0%) 1 (1%)
Intravenous rehydration (yes)	56 (59%)
BRAS	2 (2, 4)
*Range*	0–5
LOS (days)	8 (6, 10)
*Range*	3–22

* Data are reported as median (I quartile, III quartile) or range (min–max) or percentages (%). PCR = polymerase chain reaction; RSV = respiratory syncytial virus; PICU = pediatric intensive care unit; BRAS = bronchiolitis risk of admission score; LOS = length of stay; SARS-CoV-2 = Severe Acute Respiratory Syndrome Coronavirus 2.

**Table 2:** Analysis of patients admitted to PICU vs. not admitted *.

Parameters	Admission to PICU		*p*-Value °
	Yes (n = 37)	No (n = 58)	
Age (weeks)	8.3 ± 6.6	19.02 ± 12.1	**0.00**
Gender	*m* = 22; *f* = 15	*m* = 33; *f* = 25	0.80
Gestational age at birth	38.43 ± 1.96	37.94 ± 1.91	0.23
Weight at admission (gr)	4585.4 ± 1347.5	6656.2 ± 2010.2	**0.00**
Red blood cells (10^3^/μL)	3947.918 ± 563.197	4190.964 ± 564.710	**0.04**
Hemoglobin (g/dL)	12.07 ± 1.90	11.42 ± 1.21	**0.04**
White blood cells (10^3^/μL)	11.486 ± 4.575	12.050 ± 6.780	0.65
Neutrophils (10^3^/μL)	5.405 ± 2.688	5.775 ± 4.317	0.64
Lymphocytes (10^3^/μL)	5.189 ± 2.496	5.360 ± 3.698	0.80
Eosinophils (10^3^/μL)	0.173 ± 0.143	0.137 ± 0.098	0.14
Monocytes (10^3^/μL)	0.682 ± 0.337	0.775 ± 0.427	0.26
Platelets (10^3^/μL)	458.513 ± 169.951	474.068 ± 136.934	0.62
CRP (mg/dL)	0.95 ± 1.77	1.15 ± 1.71	0.59
NLR	1.20 ± 0.65	1.28 ±0.89	0.65
PLR	100.26 ± 41.74	109.63 ± 51.61	0.35
MLR	0.13 ± 0.04	0.16 ± 0.09	0.09
ELR	0.03 ± 0.02	0.02 ± 0.01	0.07
LMR	8.03 ± 2.62	7.49 ± 3.14	0.39
NPR	0.01 ± 0.006	0.01 ± 0.006	0.75
LPR	0.01 ± 0.004	0.01 ± 0.008	0.98
LNR	1.09 ± 0.58	1.23 ± 1.00	0.46
PNR	97.10 ± 39.77	114.70 ± 90.43	0.26
SII	542,177.4 ± 399,039.5	630,550.2 ± 612,431.8	0.43
SIRI	810.57 ± 628.01	1062.53 ± 1353.20	0.29
AiW/Hb	0.73 ± 0.62	1.65 ± 1.02	**0.00**
RBC/AiW/1000	744.79 ± 489.9	302.7 ± 167.05	**0.00**

* Data are reported as mean ± standard deviation, ° *p*-value by Student’s *t*-test, CRP = c-reactive protein; NLR = neutrophils/lymphocytes ratio; PLR = platelets/lymphocytes ratio; MLR = monocytes/lymphocytes ratio; ELR = eosinophils/lymphocytes ratio; LMR = lymphocytes/monocytes ratio; NPR = neutrophils/platelets ratio; LPR = lymphocytes/platelets ratio; LNR = lymphocytes/neutrophils ratio; PNR = platelets/neutrophils ratio; SII = systemic immune-inflammation index (NLR*platelets); SIRI = systemic inflammation response index (neutrophils*monocytes/lymphocytes); AiW/Hb = age in weeks/hemoglobin; RBC/AiW/1000 = red blood cells/age in weeks/1000; m = male; f = female.

**Table 3:** Analysis of significant parameters influencing PICU admission *.

Parameters	Cut-Off	Sensitivity	Specificity	PPV	NPV	OR	RR
RBCs (10^3^/μL)	≤4050	59	60	49	71	2.2	1.5
Hemoglobin (g/dL)	≥11.6	54	50	41	63	1.2	1.1
AiW/Hb	<0.85	76	74	65	83	8.9	2.9
RBC/AiW/1000	>350	78	64	58	82	6.4	2.2

* Data are reported as percentages (%) or absolute values. PPV = positive predictive value; NPV = negative predictive value; OR = odds ratio; RR = relative risk; RBCs = red blood cells; AiW/Hb = age in weeks/hemoglobin; RBC/AiW/1000 = red blood cells/age in weeks/1000.

**Table 4:** Analysis of patients who required ventilatory support vs. who not required it *.

Parameters	Ventilatory Support		*p*-Value °
	Yes (n = 62)	No (n = 33)	
Age (weeks)	11.37 ± 8.49	21.36 ± 13.62	**0.00**
Gender	*m* = 35; *f* = 27	*m* = 20; *f* = 13	0.6
Gestational age at birth	38.29 ± 1.71	37.85 ± 2.31	0.29
Weight at admission (gr)	5294.35 ± 1631.6	6893.03 ± 2339.43	**0.00**
Red blood cells (10^3^/μL)	4000.131 ± 562.635	4271.212 ± 560.004	**0.02**
Hemoglobin (g/dL)	11.62 ± 1.70	11.77 ± 1.20	0.64
White blood cells (10^3^/μL)	11.418 ± 4.235	12.606 ± 8.397	0.36
Neutrophils (10^3^/μL)	5.526 ± 2.681	5.809 ± 5.260	0.73
Lymphocytes (10^3^/μL)	5.020 ± 2.284	5.808 ± 4.586	0.26
Eosinophils (10^3^/μL)	0.154 ± 0.121	0.145 ± 0.113	0.71
Monocytes (10^3^/μL)	0.687 ± 0.294	0.836 ± 0.529	0.08
Platelets (10^3^/μL)	475.548 ± 156.802	453.848 ± 137.401	0.50
CRP (mg/dL)	1.17 ± 1.76	0.87 ± 1.68	0.42
NLR	1.28 ± 0.74	1.19 ± 0.92	0.63
PLR	108.75 ± 49.59	100.77 ± 45.12	0.44
MLR	0.15 ± 0.07	0.16 ± 0.08	0.43
ELR	0.03 ± 0.02	0.02 ±0.01	0.30
LMR	7.69 ± 2.59	7.72 ± 3.56	0.96
NPR	0.01 ± 0.006	0.01 ± 0.007	0.74
LPR	0.01 ± 0.004	0.01 ± 0.009	0.16
LNR	1.07 ± 0.65	1.37 ± 1.15	0.11
PNR	100.04 ± 43.78	122.5 ± 112.12	0.16
SII	609,983 ± 442,579	570,107 ± 691,584	0.73
SIRI	888.62 ± 712.96	1106.77 ± 1658.1	0.37
AiW/Hb	1.02 ± 0.79	1.81 ± 1.13	**0.00**
RBC/AiW/1000	552.89 ± 417.79	325.58 ± 300.66	**0.00**

* Data are reported as mean ± standard deviation. ° *p*-value by Student’s *t*-test. CRP = c-reactive protein; NLR = neutrophils/lymphocytes ratio; PLR = platelets/lymphocytes ratio; MLR = monocytes/lymphocytes ratio; ELR = eosinophils/lymphocytes ratio; LMR = lymphocytes/monocytes ratio; NPR = neutrophils/platelets ratio; LPR = lymphocytes/platelets ratio; LNR = lymphocytes/neutrophils ratio; PNR = platelets/neutrophils ratio; SII = systemic immune-inflammation index (NLR*platelets); SIRI = systemic inflammation response index (neutrophils*monocytes/lymphocytes); AiW/Hb = age in weeks/hemoglobin; RBC/AiW/1000 = red blood cells/age in weeks/1000; m = male; f = female.

**Table 5:** Analysis of significant parameters influencing ventilatory support *.

Parameters	Cut-Off	Sensitivity	Specificity	PPV	NPV	OR	RR
RBCs (10^3^/μL)	≤4300	68	52	72	46	2.2	1.4
AiW/Hb	<1	71	70	81	56	5.6	2.3
RBC/AiW/1000	>350	65	76	83	53	5.7	2.7

* Data are reported as percentages (%) or absolute values. PPV = positive predictive value; NPV = negative predictive value; OR = odds ratio; RR = relative risk; RBCs = red blood cells; RBC/AiW/1000 = age in weeks/red blood cells/1000; AiW/Hb = age in weeks/hemoglobin.

**Table 6:** Analysis of patients who required intravenous rehydration vs. who not required it *.

Parameters	Intravenous Rehydration		*p*-Value °
	Yes (n = 56)	No (n = 39)	
Age (weeks)	10.93 ± 8.94	20.46 ± 12.57	**0.00**
Gender	*m* = 31; *f* = 25	*m* = 24; *f* = 15	0.55
Gestational age at birth	38.39 ± 1.77	37.77 ± 2.12	0.12
Weight at admission (gr)	5032.14 ± 1491.34	7023.59 ± 2170.79	**0.00**
Red blood cells (10^3^/μL)	3973.418 ± 569.387	4267.179 ± 541.109	**0.01**
Hemoglobin (g/dL)	11.68 ± 1.80	11.65 ± 1.10	0.92
White blood cells (10^3^/μL)	12.425 ± 5.814	10.976 ± 6.227	0.24
Neutrophils (10^3^/μL)	5.786 ± 2.820	5.407 ± 4.826	0.63
Lymphocytes (10^3^/μL)	5.702 ± 3.746	4.707 ± 2.351	0.14
Eosinophils (10^3^/μL)	0.167 ± 0.119	0.128 ± 0.115	0.12
Monocytes (10^3^/μL)	0.750 ± 0.377	0.723 ± 0.425	0.75
Platelets (10^3^/μL)	483.357 ± 156.722	445.974 ± 138.701	0.23
CRP (mg/dL)	1.28 ± 1.85	0.76 ± 1.51	0.14
NLR	1.23 ± 0.74	1.27 ± 0.89	0.81
PLR	103.80 ± 52.16	109.12 ± 41.74	0.59
MLR	0.14 ± 0.07	0.16 ± 0.08	0.35
ELR	0.03 ± 0.01	0.02 ± 0.02	0.48
LMR	7.82 ± 2.59	7.53 ± 3.41	0.64
NPR	0.01 ± 0.006	0.01 ± 0.007	0.72
LPR	0.01 ± 0.007	0.01 ± 0.006	0.40
LNR	1.13 ± 0.69	1.24 ± 1.07	0.52
PNR	98.37 ± 44.63	121.45 ± 103.65	0.14
SII	596,060 ± 448,983	596,234 ± 652,943	0.99
SIRI	913.98 ± 733.04	1036.8 ± 1539.89	0.60
AiW/Hb	0.98 ± 0.83	1.74 ± 1.04	**0.00**
RBC/AiW/1000	593.65 ± 441.16	302.03 ± 229.06	**0.00**

* Data are reported as mean ± standard deviation. ° *p*-value by Student’s *t*-test. CRP = c-reactive protein; NLR = neutrophils/lymphocytes ratio; PLR = platelets/lymphocytes ratio; MLR = monocytes/lymphocytes ratio; ELR = eosinophils/lymphocytes ratio; LMR = lymphocytes/monocytes ratio; NPR = neutrophils/platelets ratio; LPR = lymphocytes/platelets ratio; LNR = lymphocytes/neutrophils ratio; PNR = platelets/neutrophils ratio; SII = systemic immune-inflammation index (NLR*platelets); SIRI = systemic inflammation response index (neutrophils*monocytes/lymphocytes); AiW/Hb = age in weeks/hemoglobin; RBC/AiW/1000 = red blood cells/age in weeks/1000; m = male; f = female.

**Table 7:** Analysis of significant parameters influencing intravenous rehydration *.

Parameters	Cut-Off	Sensitivity	Specificity	PPV	NPV	OR	RR
RBCs (10^3^/μL)	≤4300	71	56	70	58	3.2	1.6
AiW/Hb	<1	66	74	79	60	5.6	2.6
RBC/AiW/1000	>350	70	72	78	62	5.8	2.5

* Data are reported as percentages (%) or absolute values. PPV = positive predictive value; NPV = negative predictive value; OR = odds ratio; RR = relative risk; RBCs = red blood cells; RBC/AiW/1000 = red blood cells/age in weeks/1000; AiW/Hb = age in weeks/hemoglobin.

**Table 8:** Analysis of patients who required prolonged LOS vs. who not required it *.

Parameters	Prolonged LOS (≥6 Days)		*p*-Value °
	Yes (n = 76)	No (n = 19)	
Age (weeks)	13.81 ± 11.15	18.95 ± 12.38	0.08
Gender	*m* = 44; *f* = 32	*m* = 11; *f* = 8	1
Gestational age at birth	38.24 ± 1.95	37.73 ± 1.88	0.31
Weight at admission (gr)	5696.31 ± 2065.65	6463.16 ± 1879.87	0.14
Red blood cells (10^3^/μL)	4091.373 ± 559.861	4110.789 ± 641.262	0.89
Hemoglobin (g/dL)	11.73 ± 1.55	11.42 ± 1.52	0.43
White blood cells (10^3^/μL)	12.550 ± 6.329	8.952 ± 3.167	**0.01**
Neutrophils (10^3^/μL)	5.976 ± 4.023	4.249 ± 1.917	0.07
Lymphocytes (10^3^/μL)	5.627 ± 3.435	3.959 ± 2.074	**0.04**
Eosinophils (10^3^/μL)	0.166 ± 0.124	0.09 ± 0.061	**0.01**
Monocytes (10^3^/μL)	0.762 ± 0.419	0.646 ± 0.271	0.25
Platelets (10^3^/μL)	476.684 ± 158.223	433.315 ± 107.473	0.26
CRP (mg/dL)	1.07 ± 1.68	1.07 ± 1.96	0.99
NLR	1.23 ± 0.82	1.31 ± 0.74	0.72
PLR	98.87 ± 39.34	134.43 ± 67.19	**0.003**
MLR	0.14 ± 0.06	0.19 ± 0.11	**0.01**
ELR	0.03 ± 0.02	0.02 ± 0.01	0.17
LMR	7.96 ± 2.86	6.68 ± 3.12	0.09
NPR	0.01 ± 0.007	0.01 ± 0.004	0.11
LPR	0.01 ± 0.007	0.009 ± 0.004	0.09
LNR	1.15 ± 0.71	1.26 ± 1.33	0.61
PNR	99.67 ± 49.70	140.54 ± 133.22	**0.03**
SII	604,424 ± 575,972	562,960 ± 363,700	0.76
SIRI	988.15 ± 1222.9	869.38 ± 651.18	0.68
AiW/Hb	1.21 ± 0.98	1.63 ± 1.01	0.09
RBC/AiW/1000	521.75 ± 424.87	282.65 ± 116.78	**0.01**

* Data are reported as mean ± standard deviation. ° *p*-value by Student’s *t*-test. LOS = length of stay; CRP = c-reactive protein; NLR = neutrophils/lymphocytes ratio; PLR = platelets/lymphocytes ratio; MLR = monocytes/lymphocytes ratio; ELR = eosinophils/lymphocytes ratio; LMR = lymphocytes/monocytes ratio; NPR = neutrophils/platelets ratio; LPR = lymphocytes/platelets ratio; LNR = lymphocytes/neutrophils ratio; PNR = platelets/neutrophils ratio; SII = systemic immune-inflammation index (NLR*platelets); SIRI = systemic inflammation response index (neutrophils*monocytes/lymphocytes); AiW/Hb = age in weeks/hemoglobin; RBC/AiW/1000 = red blood cells/age in weeks/1000; m = male; f = female.

**Table 9:** Analysis of significant parameters influencing prolonged LOS *.

Parameters	Cut-Off	Sensitivity	Specificity	PPV	NPV	OR	RR
WBCs (10^3^/μL)	≥9.3	72	68	90	38	5.7	2.3
Lymphocytes (10^3^/μL)	≥4.9	57	79	91	31	4.5	2.7
Eosinophiles (10^3^/μL)	≥0.11	63	68	89	32	3.7	2.0
PLR	≤112.1	66	53	85	28	2.1	1.4
MLR	≤0.146	61	58	85	27	2.1	1.4
PNR	≤99	55	53	82	23	1.4	1.2
RBC/AiW/1000	>350	58	68	88	29	3.0	1.8

* Data are reported as percentages (%). LOS = length of stay; PPV = positive predictive value; NPV = negative predictive value; OR = odds ratio; RR = relative risk; WBCs = white blood cells; PLR = platelets/lymphocytes ratio; MLR = monocytes/lymphocytes ratio; PNR = platelets/neutrophils ratio; RBC/AiW/1000 = red blood cells/age in weeks/1000.

**Table 10:** Analysis of patients with BRAS ≥ 3 vs. BRAS ≤ 2 *.

Parameters	High BRAS (≥ 3)		*p*-Value °
	Yes (n = 40)	No (n = 55)	
Age (weeks)	8.775 ± 5.82	19.25 ± 12.64	**0.00**
Gender	*m* = 20; *f* = 20	*m* = 35; *f* = 20	0.18
Gestational age at birth	38.52 ± 1.65	37.85 ± 2.09	0.09
Weight at admission (gr)	4798.25 ± 1175.74	6614.36 ± 2203.84	**0.00**
Red blood cells (10^3^/μL)	3982.325 ± 546.127	4178.981 ± 584.095	0.10
Hemoglobin (g/dL)	12.06 ± 1.84	11.39 ± 1.23	**0.03**
White blood cells (10^3^/μL)	11.246 ± 3.7	12.255 ± 7.231	0.42
Neutrophils (10^3^/μL)	5.498 ± 2.587	5.727 ± 4.438	0.77
Lymphocytes (10^3^/μL)	4.864 ± 1.938	5.606 ± 3.956	0.27
Eosinophils (10^3^/μL)	0.145 ± 0.118	0.156 ± 0.119	0.66
Monocytes (10^3^/μL)	0.735 ± 0.336	0.741 ± 0.437	0.94
Platelets (10^3^/μL)	465.750 ± 166.233	469.654 ± 138.520	0.90
CRP (mg/dL)	1.25 ± 2.13	0.94 ± 1.37	0.39
NLR	1.31 ± 0.78	1.20 ± 0.82	0.48
PLR	108.52 ± 51.47	104.13 ± 45.71	0.66
MLR	0.16 ± 0.09	0.14 ± 0.06	0.18
ELR	0.03 ± 0.02	0.02 ± 0.01	0.66
LMR	7.35 ± 2.99	7.95 ± 2.91	0.32
NPR	0.01 ± 0.006	0.01 ± 0.007	0.89
LPR	0.01 ± 0.004	0.01 ± 0.008	0.39
LNR	1.03 ± 0.58	1.28 ± 1.02	0.17
PNR	95.47 ± 37.69	116.84 ± 92.71	0.17
SII	622,751 ± 495,218	576,772 ± 572,108	0.68
SIRI	1005.34 ± 841.33	934.63 ± 1307.3	0.76
AiW/Hb	0.76 ± 0.54	1.68 ± 1.07	**0.00**
RBC/AiW/1000	678.74 ± 477.5	324.98 ± 230.23	**0.00**

* Data are reported as mean ± standard deviation. ° *p*-value by Student’s *t*-test. BRAS = bronchiolitis risk of admission score; CRP = c-reactive protein; NLR = neutrophils/lymphocytes ratio; PLR = platelets/lymphocytes ratio; MLR = monocytes/lymphocytes ratio; ELR = eosinophils/lymphocytes ratio; LMR = lymphocytes/monocytes ratio; NPR = neutrophils/platelets ratio; LPR = lymphocytes/platelets ratio; LNR = lymphocytes/neutrophils ratio; PNR = platelets/neutrophils ratio; SII = systemic immune-inflammation index (NLR*platelets); SIRI = systemic inflammation response index (neutrophils*monocytes/lymphocytes); AiW/Hb = age in weeks/hemoglobin; RBC/AiW/1000 = red blood cells/age in weeks/1000; m = male; f = female.

**Table 11:** Analysis of significant parameters influencing BRAS ≥ 3 *.

Parameters	Cut-Off	Sensitivity	Specificity	PPV	NPV	OR	RR
Hb	>11.2	65	40	44	61	1.2	1.1
AiW/Hb	<0.85	70	73	65	77	6.2	2.6
RBC/AiW/1000	>350	78	65	62	80	6.5	2.2

* Data are reported as percentages (%). BRAS = bronchiolitis risk of admission score; PPV = positive predictive value; NPV = negative predictive value; OR = odds ratio; RR = relative risk; Hb = hemoglobin; AiW/Hb = age in weeks/hemoglobin; RBC/AiW/1000 = red blood cells/age in weeks/1000.

## Data Availability

Data are available on request from the corresponding author.

## List of Abbreviations

**Abbreviations**	**Definition**
AiW/Hb	Age in Weeks-to-Hemoglobin ratio
AUC	Area Under the Curve
BPD	Bronchopulmonary Dysplasia
BRAS	Bronchiolitis Risk of Admission Score
CBC	Complete Blood Count
CPAP	Continuous Positive Air Pressure
CRP	C-Reactive Protein
ELR	Eosinophils-to-Lymphocytes ratio
HFNC	High Flow Nasal Cannula
IQR	Interquartile Range
IV	Intravenous
LMR	Lymphocytes-to-Monocytes ratio
LNR	Lymphocytes-to-Neutrophils ratio
LOS	Length of Stay
LPR	Lymphocytes-to-Platelets ratio
LRTI	Lower Respiratory Tract Infection
MLR	Monocyte-to-Lymphocyte ratio
NIV	Non-Invasive Ventilation
NLR	Neutrophil-to-Lymphocyte ratio
NPR	Neutrophils-to-Platelets ratio
NPV	Negative Predictive Value
OR	Odds Ratio
PCR	Polymerase Chain Reaction
PICU	Pediatric Intensive Care Unit
PLR	Platelets-to-Lymphocytes ratio
PNR	Platelets-to-Neutrophils ratio
PPV	Positive Predictive Value
PW	Pediatric Ward
RBC/AiW/1000	Red blood cell-to-Age in Weeks-to-1000 ratio
RR	Relative Risk
RSV	Respiratory Syncytial Virus
SARS-CoV-2	Severe Acute Respiratory Syndrome Coronavirus 2
SD	Standard Deviation
SII	Systemic Immune-Inflammation Index
SIRI	Systemic Inflammation Response Index
